# Microbial signatures in amniotic fluid at preterm birth and association with bronchopulmonary dysplasia

**DOI:** 10.1186/s12931-023-02560-w

**Published:** 2023-10-16

**Authors:** Birte Staude, Silvia Gschwendtner, Tina Frodermann, Frank Oehmke, Thomas Kohl, Susanne Kublik, Michael Schloter, Harald Ehrhardt

**Affiliations:** 1 Department of General Pediatrics and Neonatology, Justus Liebig University and Universities of Giessen and Marburg Lung Center, Giessen, Germany; 2 German Center for Lung Research (DZL), Giessen, Germany; 3 Research Unit for Comparative Microbiome Analysis, Helmholtz Zentrum München, German Research Center for Environmental Health, Neuherberg, Germany; 4 Department of Gynecology and Obstetrics, Justus Liebig University of Giessen, Giessen, Germany; 5 German Center for Fetal Surgery and Minimally Invasive Therapy (DZFT), University of Mannheim (UMM), Mannheim, Germany; 6 Division of Neonatology and Pediatric Intensive Care Medicine, Department of Pediatrics and Adolescent Medicine, University Medical Center Ulm, Ulm, Germany

**Keywords:** Bronchopulmonary dysplasia, Preterm infant, Amniotic fluid, 16S rRNA gene, Microbiome, *Ureaplasma*, *Enterococcus*, *Escherichia*, Prospective cohort study

## Abstract

**Background:**

Microbiome dysbiosis can have long-lasting effects on our health and induce the development of various diseases. Bronchopulmonary dysplasia (BPD) is a multifactorial disease with pre- and postnatal origins including intra-amniotic infection as main risk factor. Recently, postnatal pathologic lung microbiota colonization was associated with BPD. The objectives of this prospective observational cohort study were to describe differences in bacterial signatures in the amniotic fluid (AF) of intact pregnancies without clinical signs or risk of preterm delivery and AF samples obtained during preterm deliveries and their variations between different BPD disease severity stages.

**Methods:**

AF samples were collected under sterile conditions during fetal intervention from intact pregnancies (n = 17) or immediately before preterm delivery < 32 weeks (n = 126). Metabarcoding based approaches were used for the molecular assessment of bacterial 16S rRNA genes to describe bacterial community structure.

**Results:**

The absolute amount of 16S rRNA genes was significantly increased in AF of preterm deliveries and detailed profiling revealed a reduced alpha diversity and a significant change in beta diversity with a reduced relative abundance of 16S rRNA genes indicative for *Lactobacillus* and *Acetobacter* while *Fusobacterium, Pseudomonas, Ureaplasma* and *Staphylococcus* 16S rRNA gene prevailed. Although classification of BPD by disease severity revealed equivalent absolute 16S rRNA gene abundance and alpha and beta diversity in no, mild and moderate/severe BPD groups, for some 16S rRNA genes differences were observed in AF samples. Bacterial signatures of infants with moderate/severe BPD showed predominance of 16S rRNA genes belonging to the *Escherichia-Shigella* cluster while *Ureaplasma* and *Enterococcus* species were enriched in AF samples of infants with mild BPD.

**Conclusions:**

Our study identified distinct and diverse intrauterine 16S rRNA gene patterns in preterm infants immediately before birth, differing from the 16S rRNA gene signature of intact pregnancies. The distinct 16S rRNA gene signatures at birth derive from bacteria with varying pathogenicity to the immature lung and are suited to identify preterm infants at risk. Our results emphasize the prenatal impact to the origins of BPD.

**Supplementary Information:**

The online version contains supplementary material available at 10.1186/s12931-023-02560-w.

## Background

Even more than 50 years after its original description, bronchopulmonary dysplasia (BPD) remains a highly prevalent and devastating morbidity following premature delivery [1]. The diagnosis of BPD poses a high risk of lifelong consequences for the overall health status and quality of life [2, 3]. Due to its pre- and postnatal multifactorial origins, therapeutic approaches to prevent BPD are still very restricted and of limited efficacy [4–6]. Prenatally, maternal preeclampsia/HELLP, intrauterine growth restriction and nicotine exposure constitute main risk factors. Postnatally, relevant contributors to the evolution of BPD are oxygen toxicity and mechanical ventilation. Postnatal infections aggravate the injury to the immature lung. Studies in experimental models demonstrated comparable pathomechanisms as observed during hyperoxia and mechanical ventilation [6]. Recently, microbiota colonization of the immature lung was coming into the focus of research and dysbiotic changes were observed in preterm infants after birth that later on induced BPD development [6]. However, the underlying clinical studies were of marked heterogeneity and restricted to preterm infants that required invasive mechanical ventilation where tracheal aspirates were available [7]. In these infants, a reduced alpha and beta diversity was observed, which was a result a reduced relative abundance of Lactobacilli and an increase of Enterobacteriaceae. In another study, bacterial colonization of the upper airways with *Enterococcu*s *Enterobacter Staphylococcus* and *Escherichia* species was identified to be associated with BPD [8]. But the impact of microbiota on the immature lung and the risk for BPD remains controversial [9]. The two largest meta-analyses on this topic found an association between chorioamnionitis or the pulmonary colonization with bacteria of the genus *Ureaplasma* and BPD, but both reports point to the uncertainty due to potential biases in the published data despite the statistically significant impact on BPD [10, 11]. This uncertainty is strengthened by a recent large cohort analysis where the mode of birth, which highly impacts the microbial colonization at birth, had no influence on the incidence and severity of BPD [12].

This inconsistency in evidence for the role of microbiota in BPD development stands in contrast to the results from preclinical studies that unravelled the circumstances of attenuated and aggravated lung injury in connection to the time point and mass of bacterial colonization [9]. A single prenatal lipopolysaccharide (LPS) stimulus in rodents induced BPD typical pathologies [13]. However, in foetal lambs the intrauterine colonization with *Ureaplasma* did not alter air space and vascular development despite the induction of inflammation. The clinically relevant scenario of multifactorial exposures did not provoke a pulmonary inflammatory response when intra-amniotic *Ureaplasma* application preceded the exposure to LPS [14, 15]. Comparable results were reported when antenatal steroids were applied before LPS [16]. From these studies, the term immunotolerance induction in the lung was propagated.

While the shaping of the postnatal early life microbiome has attracted particular interest [17], it is an ongoing controversial discussion whether the amniotic cavity represents a sterile environment as long as no rupture of the amniotic membranes occurs [18]. Results from molecular barcoding-based studies on amniotic fluid, placenta and foetal samples questioned the original statement of a sterile environment and quantification by realtime PCR prevailed low levels of microbial DNA. But the presence of bacterial components including their 16S rRNA gene cannot separate DNA leakage into the amniotic cavity and real living bacteria translocation [19–21].

A better understanding of the consequences of prenatal pathologies is a prerogative due to their high impact on BPD and the association of in utero microbiota structures with the pulmonary outcome in preterm infants has not been fully elaborated. In our prospective cohort study, we aimed to specify bacterial community structures in the amniotic fluid at birth in premature deliveries in comparison with intact control pregnancies and their association with the severity of BPD.

## Methods

### Study design, data acquisition and parameter definitions

Preterm infants < 32 weeks of gestation were prospectively enrolled within the PROTECT-AIRR study at the perinatal centre of the university hospital of Giessen between July 2015 and May 2020.

Data were recorded from the electronic data management system and paper file records as executed before [22]. Prenatal antibiotic therapy was counted when applied within the last 7 days before delivery. Small for gestational age (SGA) was defined as birth weight < 10th percentile of the German perinatal registry [23]. Nosocomial infections were documented applying the criteria of the German NICU nosocomial surveillance system (NEO-KISS) [24]. Preterm deliveries were categorized as intraamniotic infection whenever one of the following criteria was fulfilled: histologic chorioamnionitis, elevated AF interleukin-6 > 3600 pg/ml, intractable premature contractions under tocolytic therapy or premature rupture of membranes preceding labour.

To determine the presence and severity of BPD, the NICHD consensus definition was applied: Infants on supplemental oxygen for ≥ 28 days of life were categorized as mild BPD if they were stably breathing room air at 36 weeks postmenstrual age. Infants with persistent oxygen requirements < 30% at 36 weeks were grouped as moderate BPD and ≥ 30% of oxygen and/or need for positive pressure support as severe BPD. Therapy with highflow nasal cannulas was graded as continuous positive airway pressure whenever a level of 3-cm H2O or higher was attained and the fraction of oxygen provided by low flow nasal cannulas was calculated as published [24–26].

### Sample collection

**Fig. 1:**
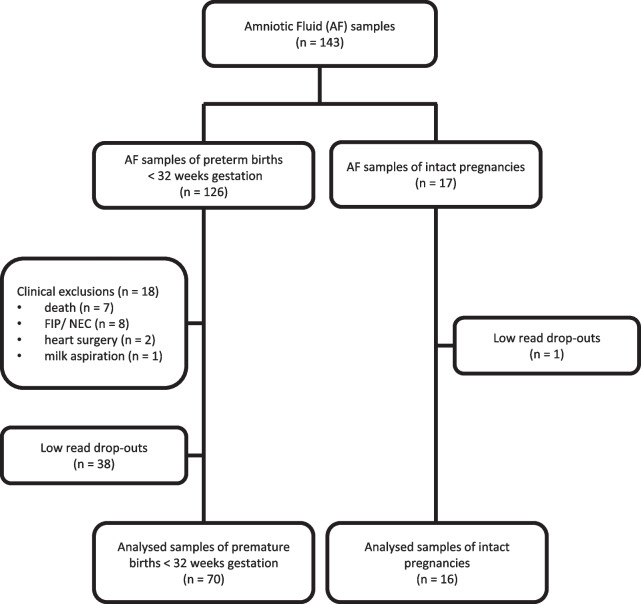
Study population flow chart. Flow chart of inclusion and exclusion of infants into the study population. Exclusion criteria included morbidities unrelated to the baseline pulmonary status but with high impact on the pulmonary outcome and AF samples with low reads bacterial signal

N = 126 infants were included into the analyses whenever an amniotic fluid (AF) sample was available (Fig. 1). AF samples from preterm deliveries were mostly obtained during routine caesarean section. In n = 4 cases samples from amniotic puncture performed immediately before the decision to premature delivery were used. n = 18 patients were excluded from the analyses due to death before 36 weeks of gestation for non-pulmonary reasons (n = 7), necrotising enterocolitis or focal intestinal perforation (n = 8), cardiac surgery (n = 2) or milk aspiration (n = 1) due to the potential high impact on the pulmonary outcome independent of the natural course (Fig. 1). n = 17 control AF samples were obtained under sterile conditions from intact pregnancies without signs for premature delivery during prenatal interventions with a median gestational age of 25 weeks that were subjected to prenatal intervention for clinical indication (Fig. 1).

The highly probable microbial contamination of samples during spontaneous delivery of preterm infants with high loads of microbiota or their particles was excluded as all AF samples were obtained immediately before delivery by amniotic puncture or during caesarean section. AF samples were collected in sterile pyrogen-free protein low-bind tubes (Eppendorf SE, Hamburg, Germany) and stored at − 80° until analysis.

### Nucleic acid extraction

1.5 mL of amniotic fluid was centrifuged for 15 min at 14,000×*g*. The obtained pellet was incubated with lysozyme (20 mg/ml) and Proteinase K (20 mg/ml) for 1 h at 37 °C. DNA was extracted following phenol–chloroform extraction protocol as described previously [27]. Additionally, blank extraction samples were performed to identify potential contamination during DNA extraction.

### Quantitative real-time PCR

Quantitative real-time PCR of the 16S rRNA gene as proxy for bacterial load was performed on the ABI 7300 Cycler (Applied Biosystems, Germany) using the following reaction mixture: 12.5 µl 2 × Power SYBR Green master mix (Thermofisher Scientific, Germany), 5 pmol primers (FP 16S/RP 16S) [43], 0.5 µl 3% BSA and 2 µl DNA template in a total volume of 25 µl. PCR conditions were 10 min at 95 °C; 40 cycles of 45 s at 95 °C, 45 s at 58 °C, 45 s at 72 °C; 10 min 72 °C; 1 cycle of 15 s at 95 °C, 30 s at 60 °C, 15 s at 95 °C. All PCR products were checked on agarose gel. Amplification efficiency (calculated by Eff = [10^(− 1/slope) − 1]) was 90% with R2 of 0.994. The quantified gene copy numbers were normalized to 1 ml of amniotic fluid.

### 16S rRNA gene sequencing

Amplicon sequencing of the V3–V4 hypervariable region of the 16S rRNA gene was performed on a MiSeq Illumina instrument (MiSeq Reagent Kit v3 (600 Cycle); Illumina, San Diego, CA, USA) using the universal eubacterial primers 347F and 803R [28], extended with sequencing adapters to match Illumina indexing primers. To identify potential contamination during DNA extraction and amplification, both extraction and PCR no template control samples were performed. PCR was done using NEBNext high fidelity polymerase (New England Biolabs, Ipswich, USA) in a total volume of 25 µl (10 ng DNA template, 12.5 µl polymerase, 5 pmol of each primer). PCR conditions were 5 min at 98 °C; 35 cycles of 10 s at 98 °C, 30 s at 56 °C, 30 s at 72 °C; 10 min 72 °C. PCR products were purified using AMPure XP beads (Beckman Coulter Life Sciences, Indianapolis, USA) and quantified via PicoGreen assay. Subsequently, indexing PCR was performed using the Nextera XT Index Kit v2 (Illumina, Inc. San Diego, CA, USA) in a total volume of 25 µl (10 ng DNA template, 12.5 µl NEBNext high fidelity polymerase, 2.5 µl of each indexing primer) and the following PCR conditions: 30s at 98 °C; 8 cycles of 10 s at 98 °C, 30s at 55 °C, 30s at 72 °C; 5min 72 °C. Indexing PCR products were purified using AMPure XP beads, qualified and quantified via a Fragment Analyzer™ instrument (Advanced Analytical Technologies, Inc., Ankeny, USA) and pooled in an equimolar ratio of 4 nM.

### Sequence data processing

Sequences were analysed using QIIME 2 software package release 2019.10.0 [29] with default parameters. FASTQ files were trimmed and merged with a minimum read length of 50 and minimum Phred score of 15 using AdapterRemoval [30]. Quality control was done using the QIIME 2 plugin DADA2 [31] by removing 20 bp n-terminally and truncating the reads at position 280 (forward) and 220 (reverse), respectively, resulting in unique amplicon sequence variants (ASV). For taxonomic analysis, Naive Bayes classifier was pre-trained on the SILVA_132_QIIME release 99% using the primer pair 347F / 803R. To exclude potential contamination, ASV occurring in extraction and PCR controls were removed from the dataset (33 and 2 ASV, respectively) (Additional file 1: Table S1). Furthermore, mitochondrial sequences and singletons were removed. Data normalization was done by subsampling to 5434 reads (lowest obtained read number). Rarefaction curves show saturation, indicating that this sampling depth was sufficient for further analysis. The sequence data obtained in this study are deposited in the short read archive of NCBI under accession number PRJNA817592.

### Statistical analysis

All statistics were performed in R version 4.0.5 (https://www.R-project.org). For qualitative parameters, Chi-Square Test and Fisher-Test were used as appropriate, with Benjamini–Hochberg p value adjustment for multiple pairwise comparison. Quantitative real-time PCR data were analyzed using Kruskal–Wallis test and Wilcoxon-rank sum test, respectively. Alpha diversity was calculated using species richness based on ASV number, Pielou’s evenness and Shannon diversity index. Beta diversity was analysed via unweighted and weighted UniFrac distance. For statistical purposes, Kruskal–Wallis test, Wilcoxon-rank sum test and PERMANOVA with Benjamini–Hochberg p value correction for multiple comparisons was used. To identify microbial taxa differing between the analysed groups, generalized linear models accounting for zero-inflation (R packages MASS and pscl) were used. For model validation, residual histograms, plots showing sample quantiles versus theoretical quantiles and plots showing residuals versus fitted values were checked for normal distribution and variance homogeneity of residuals [32]. Significant differences (p < 0.05) were calculated via ANOVA respective Wilcoxon and Kruskal–Wallis test using Benjamini–Hochberg p value adjustment for multiple pairwise comparisons. Additionally, methods for microbiome biomarker identification (LEfSe) and differential expression analysis (edgeR) were performed to verify the model results. Plots were created in R using ggplot2, ggpubr and metacoder.

## Results

### Cohort characteristics

In line with the published literature, AF samples of n = 38 infants and n = 1 control rendered low reads bacterial 16S rRNA gene signal [33] leaving n = 70 preterm infants and n = 16 intact control pregnancies for the final analyses as presented in the flowchart in Fig. 1. Of importance, no systematic bias was introduced as the low read group and the cohort available for analysis did not differ for baseline characteristics (Table 1). In our analysis cohort, the median gestational age was 28 weeks (range 23–31) and the median birth weight 970 g (range 360–1800). Gender distribution was nearly balanced with 46% females and 76% received antenatal steroids. In line with the published literature, 56% of all preterm infants in our cohort fulfilled any BPD criterion and n = 14 still depended on respiratory support and/or supplemental oxygen at 36 weeks of gestation (Table 1 presenting median and quartiles) [34].

**Table 1:** Maternal and neonatal characteristics of the total study population

	Total cohort (n = 126)	Excluded (n = 18)	p-value	Low reads (n = 38)	Included (n = 70)	p-value
Gestation [weeks]	28 [26, 29]	25 [24, 27]	< 0.001	28 [26, 29]	28 [27, 30]	0.42
Birthweight [g]	950 [700, 1293]	570 [468, 738]	< 0.001	950 [800, 1173]	970 [824, 1383]	0.27
z-score birth weight	− 0.57 [− 1.11, − 0.10]	− 1.65 [− 2.05, − 0.66]	0.0024	− 0.55 [− 1.11, 0.08]	− 0.41 [− 0.97, − 0.06]	0.57
z-score weight at 36 weeks gestational age	− 1.04 [− 1.41, − 0.54] (na = 11)	− 1.56 [− 2.05, − 0.89] (na = 6)	0.09	− 0.81 [− 1.28, − 0.81] (na = 3)	− 1.04 [− 1.36, − 0.55] (na = 2)	0.51
Δz-score (weight 36 weeks gestational age – birth)	− 0.41 [− 0.74, 0.00] (na = 11)	− 0.15 [− 0.66, 0.60] (na = 6)	0.25	− 0.30 [− 0.62, − 0.00] (na = 3)	− 0.48 [− 0.79, − 0.03] (na = 2)	0.17
Small for gestational age	26 (21%)	10 (56%)	< 0.001	8 (21%)	8 (11%)	0.29
Female	56 (45%)	5 (28%)	0.20	19 (50%)	32 (46%)	0.82
Caesarean section	122 (97%)	18 (100%)	1.00	37 (97%)	67 (96%)	1
Singleton	70 (56%)	9 (50%)	0.80	20 (53%)	41 (59%)	0.70
APGAR 5'	9 [8, 9]	8 [7, 9]	0.052	9 [8, 9]	9 [8, 9]	0.68
APGAR 10'	10 [9, 10]	9 [9, 10]	0.068	9 [9, 10]	10 [9, 10]	0.10
Maternal age	32 [27, 36]	30 [25, 34]	0.26	33 [28, 37]	33 [29, 36]	0.66
Maternal BMI at birth	30 [26, 34]	31 [26, 34]	0.43	30 [26, 34] (na = 4)	29 [26, 34] (na = 1)	0.67
Nicotine	16 (13%) (na = 2)	3 (17%)	0.70	4 (11%) (na = 1)	9 (13%) (na = 1)	0.98
Antibiotics before birth	36 (29%) (na = 4)	5 (28%)	1.00	11 (29%) (na = 3)	20 (29%) (na = 1)	0.98
Histological chorioamnionitis	25 (20%) (na = 4)	3 (17%) (na = 2)	1.00	6 (16%) (na = 1)	16 (23%) (na = 1)	0.55
Cause for premature birth			1.00			0.47
AIS	52 (41%)	7 (39%)		13 (34%)	32 (46%)	
HELLP/preeclampsia	28 (22%)	4 (22%)		11 (29%)	13 (17%)	
IUGR	20 (16%)	3 (17%)		5 (13%)	12 (17%)	
Other	26 (21%)	4 (22%)		9 (24%)	13 (19%)	
BPD	75 (60%) (na = 7)	11 (61%) (na = 7)	0.0067	25 (66%)	39 (56%)	0.42
BPD severity			< 0.001			0.47
No	44 (34%)	0 (0%)		13 (34%)	31 (44%)	
Mild	42 (33%)	3 (17%)		14 (37%)	25 (36%)	
Moderate/severe	33 (26%)	8 (44%)		11 (29%)	14 (20%)	
ANCS			0.14			0.75
None/ < 24 h	30 (24%)	4 (22%)		9 (24%)	17 (24%)	
ANCS 24 h – 7 d	68 (54%)	13 (72%)		21 (55%)	34 (49%)	
ANCS > 7 d	28 (22%)	1 (6%)		8 (21%)	19 (27%)	
ROP	51 (40%) (na = 6)	10 (56%) (na = 6)	0.0040	18 (47%)	23 (33%)	0.20
ROP treatment	13 (10%)	6 (33%)	0.011	3 (8%)	4 (6%)	0.74
PVL	4 (3%)	2 (11%)	0.097	2 (5%)	0 (0%)	0.12
IVH	8 (6%)	3 (17%)	0.087	1 (3%)	4 (6%)	0.65
Nosocomial infection	24 (19%)	9 (50%)	0.0010	8 (21%)	7 (10%)	0.20

Characteristics are separated for exclusion criteria for clinical morbidities and low read samples and included patients. Qualitative data is presented as n with proportion in brackets. Quantitative data is presented as median with 1^st^ and 3^rd^ Quartile in square brackets. Loss of data due to unavailability has been marked as “na”. For statistical analyses Wilcoxon, Chi-squared and Fisher test were used as appropriate. p-values were calculated for excluded and low-reads in comparison to the Included group respectively

*BMI* body mass index, *AIS* amnion infection syndrome, *IUGR* intrauterine growth restriction, *BPD* bronchopulmonary dysplasia, *ANCS* antenatal corticosteroids, *ROP* retinopathy of prematurity, *PVL* periventricular leukomalacia, *IVH* intraventricular haemorrhage

### Microbial signatures in intact pregnancies and preterm deliveries

**Fig. 2:**
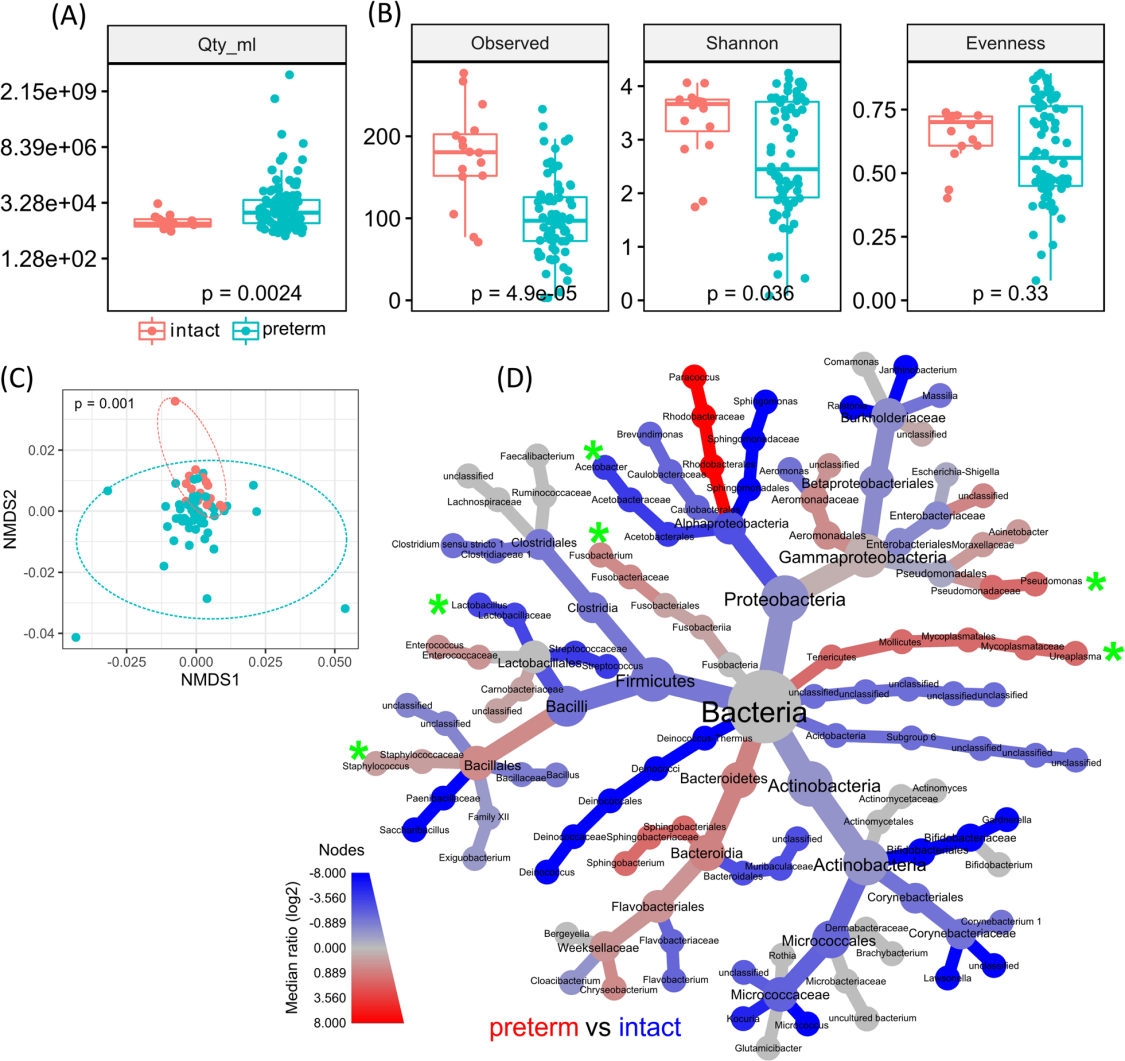
Differences in 16S rRNA gene microbial abundance in amniotic fluid samples from preterm deliveries and intact pregnancies.** A** Box plots show the gene copy numbers of 16S rRNA gene per mL of AF samples of intact pregnancy (red) and preterm delivery (blue) and prevailed a significantly higher bacterial load in AF samples of preterm deliveries. **B** Box plots of different alpha diversity indices show that richness and Shannon diversity was significantly reduced in AF samples of preterm deliveries while evenness did not differ. Statistical analysis was performed using Wilcoxon Rank-Sum test. **C** NMDS plot of weighted Unifrac distances shows significant (p < 0.05) altered bacterial 16S rRNA gene community composition for AF samples of preterm deliveries. Statistical analysis was performed using PERMANOVA with Benjamini–Hochberg correction for multiple comparisons. **D** Heat tree including genera ≥ 0.1% of all reads. The tree shows the taxonomic information (domain to genus) and represents a comparison between preterm AF samples and samples of intact pregnancies. Coloured taxa are more abundant (based on log2-transformed ratio of median proportions) in the samples indicated (red: increased in preterm, blue: increased in intact). Significant changes (p < 0.05 based on Wilcoxon-Rank-Sum test with FDR correction) are marked with asterisks

Comparing intact pregnancies and preterm deliveries, bacterial load in the AF was significantly higher whereas the alpha diversity was reduced in the preterm deliveries (Fig. 2A, B). Beta diversity showed clearly differences in 16S rRNA gene composition for preterm AF samples (Fig. 2C), with 16S rRNA gene sequences indicative for genera belonging to *Proteobacteria*, *Firmicutes, Fusobacteria* and *Tenericutes* differing significantly between AF samples of preterms and intact pregnancies. While ASV assigned to *Acetobacter* (*Alphaproteobacteria*) and *Lactobacillus* (*Firmicutes*) were significantly reduced in preterm AF samples, *Pseudomonas* (*Gammaproteobacteria*), *Staphylococcus* (*Firmicutes*), *Fusobacterium* (*Fusobacteria*) and *Ureaplasma* (*Tenericutes*) prevailed increased in relative 16S rRNA gene abundance (Fig. 2D). These results reveal for the first time a lower 16S rRNA gene load and higher richness in the AF of intact pregnancies compared to preterm deliveries.

### Microbial signatures separated by BPD disease severity stages

To study the association of specific microbial 16S rRNA gene signatures in the AF at birth with the later development of BPD, infants were separated by the severity of BPD into no (n = 31), mild (n = 25) and moderate/severe (n = 14) BPD cases where the last category comprises the most severely affected infants with the highest risk for lifetime pulmonary sequelae. As expected, infants within the mild and moderate/severe BPD categories were more immature and had a lower gestational age at birth than infants within the no BPD category while they did not differ for maternal characteristics (Table 2). No significant differences in baseline parameters became evident between the two groups of infants fulfilling the BPD criterion besides lower birth weight (p = 0.045) (Table 2). Fitting to the other respiratory variables, the maximum fraction of oxygen required to meet the oxygen saturation target was significantly increased within the first 72 h in infants with moderate/severe BPD reflecting the more compromised gas exchange at birth (Table 2).

**Table 2:** Neonatal characteristics of patients included in the analyses separated for the severity of BPD

	No BPD (n = 31)	Mild BPD (n = 25)	Morderate/severe BPD (n = 14)	p-value
Weeks	30 [29, 30]	27 [26, 28]*	25 [24, 27]*^,†^	< 0.001
Birth weight [g]	1390 [1140, 1475]	885 [800, 970]*	695 [575, 879]*^,†^	< 0.001
z-score birth weight	− 0.15 [− 0.56, 0.01]	− 0.58 [− 0.88, − 0.23]	− 0.90 [− 1.25, − 0.42]^*^	0.0094
z-score weight at 36 weeks gestational age	− 0.80 [− 1.11, − 0.46] (na = 1)	− 1.02 [− 1.36, − 0.62] (na = 1)	− 1.32 [− 1.55, − 1.09]*	0.025
Δz-score (weight 36 weeks gestational age–birth)	− 0.49 [− 0.94, 0.04] (na = 1)	− 0.50 [− 0.75, − 0.30] (na = 1)	− 0.41 [− 0.74, − 0.25]	0.99
Small for gestational age	3 (10%)	2 (8%)	3 (21%)	0.49
Female	13 (42%)	13 (52%)	6 (43%)	0.78
Caesarean section	30 (97%)	24 (96%)	13 (93%)	0.79
Singleton	17 (55%)	14 (56%)	10 (71%)	0.57
APGAR 5'	9 [8, 9]	9 [8, 9]	9 [8, 9]	0.76
APGAR 10'	10 [9, 10]	10 [9, 10]	10 [9, 10]	0.50
Maternal age	33 [29, 34]	33 [25, 37]	33 [26, 36]	0.90
Maternal BMI at birth	29 [28, 37]	29 [26, 32] (na = 1)	29 [25, 32]	0.54
Nicotine	5 (16%) (na = 1)	3 (12%)	1 (7%)	0.73
Antibiotics before birth	9 (29%)	5 (20%) (na = 1)	6 (43%)	0.35
Histological chorioamnionitis	7 (23%)	4 (16%)	5 (36%) (na = 1)	0.30
Cause for premature birth				0.82
AIS	16 (52%)	9 (36%)	7 (50%)	
HELLP/preeclampsia	4 (13%)	7 (28%)	2 (14%)	
IUGR	6 (19%)	4 (16%)	2 (14%)	
Other	5 (16%)	5 (20%)	3 (21%)	
ANCS				0.073
None/ < 24 h	10 (32%)	5 (20%)	2 (14%)	
ANCS 24 h – 7 d	10 (32%)	13 (52%)	11 (79%)	
ANCS > 7 d	11 (35%)	7 (28%)	1 (7%)	
ROP	3(10%)	10 (40%)*	10 (71%)*	< 0.001
ROP treatment	0 (0%)	0 (0%)	4 (29%)	0.25
PVL	0 (0%)	0 (0%)	0 (0%)	1
IVH	1 (3%)	1 (4%)	2 (14%)	0.33
Pneumothorax	1 (3%)	0 (0%)	0 (0%)	0.60
Nosocomial infection	0 (0%)	1 (4%)	6 (42%)*^,†^	< 0.001
Duration invasive ventilation [d]	0 [0, 0]	0 [0,2]*	0 [0, 22]*	0.0076
Duration non-invasive ventilation [d]	8 [5, 22]	42 [32, 49]*	67 [49, 97]*^,†^	< 0.001
Oxygen supplementation at discharge	0 (0%)	0 (0%)	2 (14%)	0.033
Max. FiO_2_ (24 h)	0.30 [0.24, 0.38]	0.34 [0.30, 0.42]	0.39 [0.33, 0.54]	0.060
Intubation within 24 h	3 (10%)	6 (24%)	6 (42%)	0.042
Max. invasive PIP (24 h) [cmH_2_0]	16 [15, 18] (na = 27)	15 [15, 17] (na = 18)	16 [8, 16] (na = 9)	0.81
Max. invasive PEEP (24 h) [cmH_2_O]	6 [6, 7] (na = 27)	6 [6, 7] (na = 18)	6 [6] (na = 9)	0.63
Max. invasive MAP (24 h) [cmH_2_O]	9 [9, 10] (na = 28)	9 [8, 9] (na = 19)	9 [8, 9] (na = 9)	0.86
Max. FiO_2_ (72 h)	30 [24, 38]	36 [30, 50]*	40 [36, 54]*	0.022
Intubation within 72 h	3 (10%)	9 (36%)	6 (42%)	0.017
Max. invasive PIP (72 h) [cmH_2_0]	18 [16, 21] (na = 27)	16 [15, 17] (na = 16)	17 [16, 18] (na = 9)	0.35
Max. invasive PEEP (72 h) [cmH_2_O]	6 [6, 7] (na = 27)	6 [6] (na = 16)	6 [6] (na = 9)	0.34
Max. invasive MAP (72 h) [cmH_2_O]	9 [9, 10] (na = 28)	9 [8, 10] (na = 17)	9 [8, 10] (na = 9)	0.79
Surfactant	10 (32%)	19 (76%)*	11 (79%)*	< 0.001
Surfactant doses (72 h)	0 [0, 1]	1 [1]*	1 [1]*	< 0.001
Postnatal corticosteroids	0 (0%)	1 (4%)	2 (14%)	0.048

Qualitative data is presented as n with proportion in brackets. Quantitative data is presented as median with 1st and 3rd quartile in square brackets. Not available data has been marked as “na”. For statistical analyses Kruskal–Wallis and Fisher test were used as appropriate. p-values indicate differences between all three groups. For post-hoc-analyses Wilcoxon pairwise comparison and Fisher test were used with Benjamini–Hochberg adjusting for multivariate pairwise comparison

*BMI* body mass index, *AIS* amnion infection syndrome, *IUGR* intrauterine growth restriction, *BPD* bronchopulmonary dysplasia, *ANCS* antenatal corticosteroids, *ROP* retinopathy of prematurity, *PVL* periventricular leukomalacia, *IVH* intraventricular haemorrhage, *FiO2* fraction of inspired oxygen, *PIP* positive inspiratory pressure, *PEEP* positive endexpiratory pressure, *MAP* mean airway pressure

*Indicates a difference to the no BPD group with p < 0.05, † indicates a difference to the BPD 1 group with p < 0.05

**Fig. 3:**
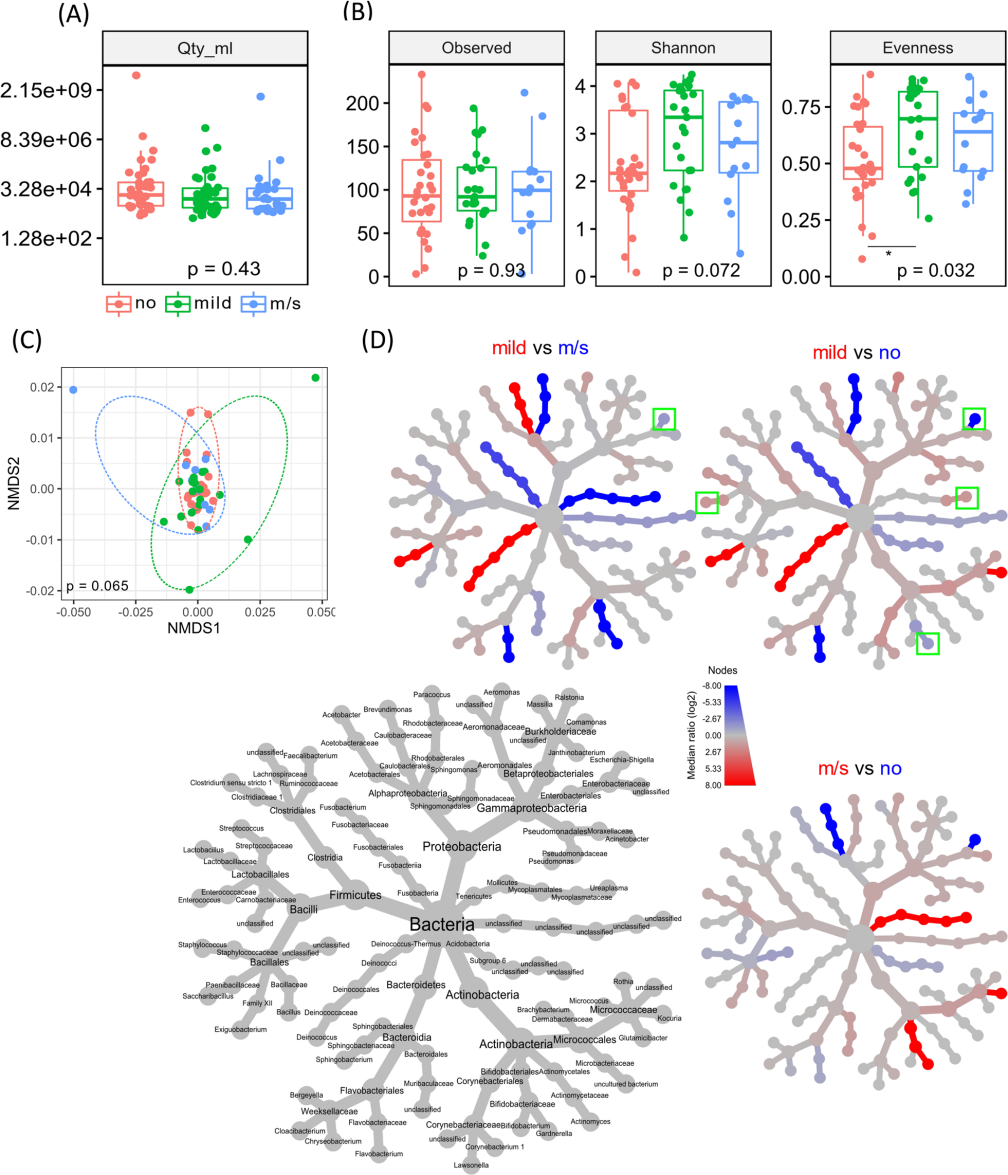
Disparities in relative but not absolute 16S rRNA gene microbial abundance in amniotic fluid samples from preterm deliveries depending on the BPD disease severity. **A** AF samples from preterm deliveries from Fig. 2 were separated for the BPD disease severity into no (red), mild (green) and moderate/severe (m/s, blue) BPD. Box plots show the gene copy numbers of 16S rRNA gene per ml of AF samples and prevailed no significant difference in total bacterial load. Statistical analysis was performed using Kruskal–Wallis and Wilcoxon Rank-Sum test with Benjamini–Hochberg correction for multiple comparisons, respectively. **B** Box plots of different alpha (diversity indices show that richness and Shannon diversity did not differ significantly between the samples of different disease severities while evenness of mild BPD was increased. Statistical analysis was performed using Kruskal–Wallis and Wilcoxon Rank-Sum test with Benjamini–Hochberg correction for multiple comparisons, respectively. **C** NMDS plot of weighted Unifrac distances shows no significant (p < 0.05) difference in bacterial 16S rRNA gene community composition for samples of different disease severities. Statistical analysis was performed using PERMANOVA with Benjamini–Hochberg correction for multiple comparisons. **D** Heat tree including genera ≥ 0.1% of all reads. The labelled tree on the lower left shows the taxonomic information (domain to genus) and is the key for the unlabelled smaller trees. Smaller trees represent a pairwise comparison between no, mild and moderate/severe (m/s) BPD. Coloured taxa are more abundant (based on log2-transformed ratio of median proportions) in the samples indicated in the respective tree subtitle colour. Significant changes (p < 0.05 based on Wilcoxon-Rank-Sum test with FDR correction) are marked with green rectangles

Total bacterial 16S rRNA gene load, gene alpha and beta diversity did not differ between the three BPD categories (except partly increased evenness of mild BPD) arguing for comparable absolute abundance and diversity pattern of bacterial taxa (Fig. 3A–C). However, at the level of single ASV, 16S rRNA sequences indicative for *Enterococcus* (*Firmicutes*) and *Ureaplasma* (*Tenericutes*) were present in higher relative abundance in the mild BPD compared to the no BPD group while 16S rRNA genes of *Gardnerella* (*Actinobacteria*) and members of the *Escherichia*-*Shigella* complex (*Proteobacteria*) displayed a significantly lower relative abundance (Fig. 3D). When infants with moderate/severe BPD were analysed in comparison to the mild BPD group, relative abundance of *Proteobacteria* within the *Escherichia*-*Shigella* group was significantly higher in the moderate/severe BPD cases while no significant variations were present for the further ASV that differed between infants with no and mild BPD (Fig. 3D). These species did as well not differ significantly in relative abundance between the no and the moderate/severe category. LEfSe analysis revealed LDA scores of 4.1–5.2 (p < 0.05), confirming those taxa being relevant biomarkers for the respective groups.

## Discussion

### Distinct 16S rRNA genes composition in intact pregnancies and preterm deliveries

Our study demonstrates that loads as well as composition of 16S rRNA genes differ between prematurely born infants immediately before their deliveries and intact pregnancies of comparable gestational age. The absolute 16S rRNA gene abundance was increased in preterm deliveries while alpha diversity was decreased and beta diversity showed a significantly altered microbial community composition, arguing for important differences in microbial 16S rRNA gene abundance and composition. Our data are congruent with previous publications, where a higher vaginal bacterial load and a *Lactobacillus* poor bacterial community with the predominance of *Ureaplasm*a and *Fusobacterium* were associated with preterm birth [35, 36]. The results of our AF samples indicate similar changes in AF bacterial DNA signatures immediately before premature delivery but it might be possible that further taxa including *Acetobacter*, *Staphylococcus* and *Pseudomonas* need to be considered. Therefore our association study might be suited to identify infants at risk for preterm delivery already before birth.

### Separation of BPD disease severity stages by 16S rRNA genes at birth

Of highest relevance, our study provides for the first-time conclusive evidence that distinct AF bacterial 16S rRNA gene signatures immediately before delivery separate infants for different BPD disease severities. In contrast to the results for premature delivery, here neither the total 16S rRNA gene abundance nor the alpha and beta diversity segregated BPD categories but differences in ASV linked to the relative abundance of specific genera were observed. In AF samples of infants with mild BPD, ASV assigned to *Ureaplasma* and *Enterococcus* species were increased compared to no BPD samples. While comparable trends were observed in infants with moderate/severe BPD, ASV linked to the *Escherichia-Shigella* complex significantly differ between mild and moderate/severe BPD samples with *Escherichia-Shigella* being increased in the latter group. By reporting an association of prenatal AF 16S rRNA gene-based microbial community changes and BPD severity, the presented data are unique and in line with recent association studies on the consequences of aberrant microbial lung colonization after birth or postnatal infections [37, 38]. Two recent publications provided conclusive evidence, that changes in the vaginal microbiome and in the airway microbiome drive innate immune activation towards an inflammatory status of the metabolome [37, 39]. Although not investigated here, a similar connection between a dysbiotic structure of the AF microbiome and metabolomic alterations might be assumed and explain the prenatal inflammatory damage to the immature lung.

### Results of the study in the context of published preclinical studies

Our clinical associations are solidified by a well-founded level of preclinical evidence where causal relations between microbial actions and BPD development were studied on an unprecedented level: Both prenatal and postnatal LPS exposure derived from *Escherichia coli* caused the typical severe hallmarks of BPD. The LPS effects on the immature lung were more pronounced with the increased dosage of LPS. Furthermore, lung injury could be worsened by synergistic effects of LPS and hyperoxia [13, 40]. Further elaboration of the model separated effects on the lung that were induced both by LPS or hyperoxia alone including exhaustion of antioxidative capacities while cell proliferation and vascular development and function were particularly affected during the combined exposure. Probably most important, the authors were able to ascribe adaptive immune responses to low dosages of LPS, and here subsequent hyperoxia did not aggravate lung injury. In contrast, high dosages of LPS plus hyperoxia caused the worst BPD phenotype that mimics the most frequent clinical scenario of excessive intraamniotic infection and subsequent need for postnatal supplemental oxygen [41]. Lastly and in line with the previous data, the germ-free conditions in gnotobiotic mice attenuated the pathologies of BPD provoked by hyperoxia, particularly inflammation that were traced back to the lack of preceding shaping of the host immune system [42]. Vice versa, prenatal antibiotic therapy disrupted the intestinal bacterial colonization of the offspring with commensals resulting in a more severe lung injury in the newborn hyperoxia mouse model [43]. When taking all these results into consideration, our results on the disparate microbial 16S rRNA gene signatures in infants with mild and moderate/severe BPD are not surprising and cannot be traced back solely to disparities in clinical baseline parameters including gestational age and birth weight. Here, the AF samples from intact pregnancies of equivalent gestational age as the moderate/severe BPD group substantiate our conclusions. *Ureaplasma* and most likely as well *Enterococcus* induce a restricted pro-inflammatory response or even an immunotolerance phenotype in animal studies [15, 40]. A similar phenomenon although not that pronounced was observed in the immune cells of premature infants [44]. In contrast, the overwhelming effects of bacteria of the *Escherichia-Shigella* complex with the release of LPS can induce a pronounced inflammatory response and lung injury during severe intraamniotic infection [41]. Interestingly, bacteria of the *Escherichia-Shigella* complex were also highly abundant in the group of infants without BPD. Here, the five weeks older gestational age at birth and the concomitant tremendous differences in maturity of the lung and the immune system may account for the absent predisposition for severe lung injury. It needs to be mentioned that *Paracoccus* was present in about half of samples studied. Although mainly present in diverse natural environments like soil, pristine or marine sediments, members of this genus could have been isolated from human skin or eye, too, and were implicated in opportunistic infections of humans [45, 46]. However, its relative proportion was < 0.5% except for 3 samples and is therefore not of relevance to our results.

### Limitations of the study

We need to acknowledge several limitations of our study. Most importantly, we did not investigate living bacteria but executed 16S rRNA gene-based sequencing in our study. Parallel microbial cultivation was not feasible as all pregnant women with preterm labour and premature rupture of membranes received antibiotics before delivery. Repeated sampling was not feasible as decision to preterm delivery and birth intervals were mostly short and additional prenatal sample collection by amniotic puncture not justifiable for ethical reasons. Thereby, we are unable to allocate the origin of the 16S rRNA genes to transmigration of living bacteria that are rapidly killed or leakage of bacterial particles as described in septic patients and healthy controls before [47–50]. Particles might derive from the gut, oropharynx or other microbial niches of the body and transmission might be propagated by maternal local or systemic inflammation in our setting [35, 51, 52]. Taking all these considerations into account, the bacterial 16S rRNA gene transmission might be caused by maternal inflammation and accompany that of inflammatory particles into the amniotic cavity. Due to the nature of prematurity, some AF samples rendered low reads of bacterial 16S rRNA genes using appropriate sequencing techniques [50] and did not meet the threshold of > 5434 bacterial reads (which was taken to cover the microbial diversity within the sample based on rarefaction curve analysis) [33] preventing analysis of all samples collected. A systematic bias was excluded as cohort characteristics did not differ between excluded and included patients (Table 1). Furthermore, we cannot completely exclude sampling contamination. But such selective contamination of the sampling environment of the abdominal skin within one isolated surgery room used are unlikely. These limitations need to be carefully addressed in subsequent studies to clarify the presence of a livid intra-amniotic microbiome and its origin. The restriction in sample size derives from the single centre approach and is too low to further separate beyond the severity stage of BPD. Therefore, we were unable to respect the causes of preterm delivery, particularly for intraamniotic infection. This needs to be addressed within future larger scaled prospective multicentre studies. Furthermore, the number of samples available for analyses from intact pregnancies (n = 16) compared to the AF samples of preterm deliveries (n = 70) and the fraction of preterm infants developing moderate/severe BPD (n = 14 of n = 70) was limited.

Our cohort displays heterogenous baseline characteristics between infants without and with BPD. The high disparities for the gestational age at birth derive from the nature of BPD as immaturity of the lung constitutes the main risk factor. But it needs to be stated that nearly all very immature infants < 28 weeks of gestation develop more or less pronounced restrictions in lung function. Therefore, the focus on the comparison of mild and moderate/severe BPD cases is of higher relevance. The addition of AF samples from control pregnancies might expand our knowledge of our study on the differences in 16S rRNA genes [53]. Our data argue towards focussing on disease severities of BPD instead of comparing the yes/no criterion that compares infants of completely different prerequisites. From all these considerations it becomes clear that our data are not suited to specify the causality between *Ureaplasma* or *Enterococcus* colonization and the outcome of mild restrictions in lung function, but a more severe BPD disease severity in connection to *Escherichia-Shigella* species.

## Conclusions

Overall, our results provide first-time evidence that microbial 16S rRNA gene signatures in the AF at birth differ between BPD disease categories. These data have the potential to guide future research directions towards the prenatal origins of BPD. The clear distinction of 16S rRNA gene microbiome signatures between intact pregnancies and preterm deliveries and the association with BPD severity categories in a limited number of participants argues towards a relevant impact on BPD. Future studies need to specify whether microbiota shaping therapies or immunomodulatory properties constitute appropriate therapeutic approaches or whether both aspects need to be considered as it is probably the case for the highly beneficial effects of breast milk to prevent BPD [54, 55]. A detailed knowledge on the mutual interference of microbiome structures and the immune system in the clinical arena can deliver the basis to bridge the insights from preclinical studies to the clinics and to direct future research strategies to prevent BPD. Our results argue that it might be necessary to initiate such a strategy before delivery. Finally, our results of distinct 16S rRNA gene-based microbiota signatures in different BPD disease categories demand such evaluation within further lung diseases and beyond and might give rise to a novel mechanistic concept of drivers of disease severity.

### Supplementary Information

**Additional file 1**:** Table S1.** List of Amplicon sequence variants (ASV) detected in blanks. Those ASV were considered as potential contaminants and removed from AF dataset before analysis.

## Data Availability

The data that support the findings of this study are available on reasonable request from the corresponding author.

## Abbreviations

BPD: Bronchopulmonary dysplasia
AF: Amniotic fluid
LPS: Lipopolysaccharide
SGA: Small for gestational age
ASV: Amplicon sequence variant

## References

[1] Northway WH, Rosan RC, Porter DY (1967). Pulmonary disease following respirator therapy of hyaline-membrane disease. Bronchopulmonary dysplasia. N Engl J Med.

[2] Bårdsen T, Røksund OD, Benestad MR, Hufthammer KO, Clemm HH, Mikalsen IB (2022). Tracking of lung function from 10 to 35 years after being born extremely preterm or with extremely low birth weight. Thorax.

[3] Lee DMX, Tan AKS, Ng YPM, Amin Z (2023). Quality of life of patients and caregivers affected by bronchopulmonary dysplasia: a systematic review. Qual Life Res.

[4] Laughon MM, Langer JC, Bose CL, Smith PB, Ambalavanan N, Kennedy KA (2011). Prediction of bronchopulmonary dysplasia by postnatal age in extremely premature infants. Am J Respir Crit Care Med.

[5] Schmidt B, Roberts R, Millar D, Kirpalani H (2008). Evidence-based neonatal drug therapy for prevention of bronchopulmonary dysplasia in very-low-birth-weight infants. Neonatology.

[6] Holzfurtner L, Shahzad T, Dong Y, Rekers L, Selting A, Staude B (2022). When inflammation meets lung development—an update on the pathogenesis of bronchopulmonary dysplasia. Mol Cell Pediatr..

[7] Pammi M, Lal CV, Wagner BD, Mourani PM, Lohmann P, Luna RA (2019). Airway microbiome and development of bronchopulmonary dysplasia in preterm infants: a systematic review. J Pediatr.

[8] Lauer T, Behnke J, Oehmke F, Baecker J, Gentil K, Chakraborty T (2020). Bacterial colonization within the first six weeks of life and pulmonary outcome in preterm infants <1000 g. J Clin Med.

[9] Dong Y, Rivetti S, Lingampally A, Tacke S, Kojonazarov B, Bellusci S, Ehrhardt H (2022). Insights into the black box of intra-amniotic infection and its impact on the premature lung: from clinical and preclinical perspectives. Int J Mol Sci.

[10] Lowe J, Watkins WJ, Edwards MO, Spiller OB, Jacqz-Aigrain E, Kotecha SJ, Kotecha S (2014). Association between pulmonary ureaplasma colonization and bronchopulmonary dysplasia in preterm infants: updated systematic review and meta-analysis. Pediatr Infect Dis J.

[11] Hartling L, Liang Y, Lacaze-Masmonteil T (2012). Chorioamnionitis as a risk factor for bronchopulmonary dysplasia: a systematic review and meta-analysis. Arch Dis Child Fetal Neonatal Ed.

[12] Ehrhardt H, Desplanches T, van Heijst A, Toome L, Fenton A, Torchin H (2022). Mode of delivery and incidence of bronchopulmonary dysplasia: results from the population-based EPICE cohort. Neonatology.

[13] Stranik J, Kacerovsky M, Vescicik P, Faist T, Jacobsson B, Musilova I (2022). A rodent model of intra-amniotic inflammation/infection, induced by the administration of inflammatory agent in a gestational sac, associated with preterm delivery: a systematic review. J Matern Fetal Neonatal Med.

[14] Polglase GR, Dalton RGB, Nitsos I, Knox CL, Pillow JJ, Jobe AH (2010). Pulmonary vascular and alveolar development in preterm lambs chronically colonized with Ureaplasma parvum. Am J Physiol Lung Cell Mol Physiol.

[15] Kallapur SG, Kramer BW, Knox CL, Berry CA, Collins JJP, Kemp MW (2011). Chronic fetal exposure to Ureaplasma parvum suppresses innate immune responses in sheep. J Immunol.

[16] Kuypers E, Collins JJP, Kramer BW, Ofman G, Nitsos I, Pillow JJ (2012). Intra-amniotic LPS and antenatal betamethasone: inflammation and maturation in preterm lamb lungs. Am J Physiol Lung Cell Mol Physiol.

[17] Stiemsma LT, Michels KB (2018). The role of the microbiome in the developmental origins of health and disease. Pediatrics.

[18] Blaser MJ, Devkota S, McCoy KD, Relman DA, Yassour M, Young VB (2021). Lessons learned from the prenatal microbiome controversy. Microbiome.

[19] de Goffau MC, Lager S, Sovio U, Gaccioli F, Cook E, Peacock SJ (2019). Human placenta has no microbiome but can contain potential pathogens. Nature.

[20] Leiby JS, McCormick K, Sherrill-Mix S, Clarke EL, Kessler LR, Taylor LJ (2018). Lack of detection of a human placenta microbiome in samples from preterm and term deliveries. Microbiome.

[21] Sterpu I, Fransson E, Hugerth LW, Du J, Pereira M, Cheng L (2021). No evidence for a placental microbiome in human pregnancies at term. Am J Obstet Gynecol.

[22] Behnke J, Estreich V, Oehmke F, Zimmer K-P, Windhorst A, Ehrhardt H (2022). Compatibility of rapid enteral feeding advances and noninvasive ventilation in preterm infants—an observational study. Pediatr Pulmonol.

[23] Voigt M, Schneider KT, Jährig K (1996). Analyse des Geburtengutes des Jahrgangs 1992 der Bundesrepublik Deutschland. Teil 1: Neue Perzentilwerte für die Körpermasse von Neugeborenen. [Analysis of a 1992 birth sample in Germany. 1: New percentile values of the body weight of newborn infants]. Geburtshilfe Frauenheilkd..

[24] Thiess T, Lauer T, Woesler A, Neusius J, Stehle S, Zimmer K-P (2021). Correlation of early nutritional supply and development of bronchopulmonary dysplasia in preterm infants <1,000 g. Front Pediatr.

[25] Wilkinson DJ, Andersen CC, Smith K, Holberton J (2008). Pharyngeal pressure with high-flow nasal cannulae in premature infants. J Perinatol.

[26] Walsh M, Engle W, Laptook A, Kazzi SNJ, Buchter S, Rasmussen M, Yao Q (2005). Oxygen delivery through nasal cannulae to preterm infants: can practice be improved?. Pediatrics.

[27] Lueders T, Manefield M, Friedrich MW (2004). Enhanced sensitivity of DNA- and rRNA-based stable isotope probing by fractionation and quantitative analysis of isopycnic centrifugation gradients. Environ Microbiol.

[28] Nossa CW, Oberdorf WE, Yang L, Aas JA, Paster BJ, Desantis TZ (2010). Design of 16S rRNA gene primers for 454 pyrosequencing of the human foregut microbiome. World J Gastroenterol.

[29] Caporaso JG, Kuczynski J, Stombaugh J, Bittinger K, Bushman FD, Costello EK (2010). QIIME allows analysis of high-throughput community sequencing data. Nat Methods.

[30] Schubert M, Lindgreen S, Orlando L (2016). AdapterRemoval v2: rapid adapter trimming, identification, and read merging. BMC Res Notes.

[31] Callahan BJ, McMurdie PJ, Rosen MJ, Han AW, Johnson AJA, Holmes SP (2016). DADA2: High-resolution sample inference from Illumina amplicon data. Nat Methods.

[32] Rosenblad AJJ (2009). Faraway: extending the linear model with R: generalized linear, mixed effects and nonparametric regression models. Comput Stat.

[33] Gallacher D, Mitchell E, Alber D, Wach R, Klein N, Marchesi JR, Kotecha S (2020). Dissimilarity of the gut–lung axis and dysbiosis of the lower airways in ventilated preterm infants. Eur Respir J.

[34] Shah PS, Lui K, Sjörs G, Mirea L, Reichman B, Adams M (2016). Neonatal outcomes of very low birth weight and very preterm neonates: an international comparison. J Pediatr.

[35] Staude B, Oehmke F, Lauer T, Behnke J, Göpel W, Schloter M (2018). The microbiome and preterm birth: a change in paradigm with profound implications for pathophysiologic concepts and novel therapeutic strategies. Biomed Res Int.

[36] DiGiulio DB, Callahan BJ, McMurdie PJ, Costello EK, Lyell DJ, Robaczewska A (2015). Temporal and spatial variation of the human microbiota during pregnancy. Proc Natl Acad Sci USA.

[37] Lal CV, Kandasamy J, Dolma K, Ramani M, Kumar R, Wilson L (2018). Early airway microbial metagenomic and metabolomic signatures are associated with development of severe bronchopulmonary dysplasia. Am J Physiol Lung Cell Mol Physiol.

[38] Jung E, Lee BS (2019). Late-onset sepsis as a risk factor for bronchopulmonary dysplasia in extremely low birth weight infants: a nationwide cohort study. Sci Rep.

[39] Pruski P, Correia GDS, Lewis HV, Capuccini K, Inglese P, Chan D (2021). Direct on-swab metabolic profiling of vaginal microbiome host interactions during pregnancy and preterm birth. Nat Commun.

[40] Shrestha AK, Bettini ML, Menon RT, Gopal VYN, Huang S, Edwards DP (2019). Consequences of early postnatal lipopolysaccharide exposure on developing lungs in mice. Am J Physiol Lung Cell Mol Physiol.

[41] Shrestha AK, Menon RT, El-Saie A, Barrios R, Reynolds C, Shivanna B (2020). Interactive and independent effects of early lipopolysaccharide and hyperoxia exposure on developing murine lungs. Am J Physiol Lung Cell Mol Physiol.

[42] Dolma K, Freeman AE, Rezonzew G, Payne GA, Xu X, Jilling T (2019). Effects of hyperoxia on alveolar and pulmonary vascular development in germ-free mice. Am J Physiol Lung Cell Mol Physiol.

[43] Willis KA, Siefker DT, Aziz MM, White CT, Mussarat N, Gomes CK (2020). Perinatal maternal antibiotic exposure augments lung injury in offspring in experimental bronchopulmonary dysplasia. Am J Physiol Lung Cell Mol Physiol.

[44] Azizia M, Lloyd J, Allen M, Klein N, Peebles D (2012). Immune status in very preterm neonates. Pediatrics.

[45] Dong K, Pu J, Yang J, Zhou G, Gao Y, Kang Z (2023). Whole-genome sequencing of Paracoccus species isolated from the healthy human eye and description of *Paracoccus shanxieyensis* sp. nov. Int J Syst Evol Microbiol..

[46] Lasek R, Szuplewska M, Mitura M, Decewicz P, Chmielowska C, Pawłot A (2018). Genome structure of the opportunistic pathogen paracoccus yeei (Alphaproteobacteria) and identification of putative virulence factors. Front Microbiol.

[47] Gosiewski T, Ludwig-Galezowska AH, Huminska K, Sroka-Oleksiak A, Radkowski P, Salamon D (2017). Comprehensive detection and identification of bacterial DNA in the blood of patients with sepsis and healthy volunteers using next-generation sequencing method—the observation of DNAemia. Eur J Clin Microbiol Infect Dis.

[48] Wang X, Buhimschi CS, Temoin S, Bhandari V, Han YW, Buhimschi IA (2013). Comparative microbial analysis of paired amniotic fluid and cord blood from pregnancies complicated by preterm birth and early-onset neonatal sepsis. PLoS ONE.

[49] Lim ES, Rodriguez C, Holtz LR (2018). Amniotic fluid from healthy term pregnancies does not harbor a detectable microbial community. Microbiome.

[50] Urushiyama D, Suda W, Ohnishi E, Araki R, Kiyoshima C, Kurakazu M (2017). Microbiome profile of the amniotic fluid as a predictive biomarker of perinatal outcome. Sci Rep.

[51] Cobo T, Vergara A, Collado MC, Casals-Pascual C, Herreros E, Bosch J (2019). Characterization of vaginal microbiota in women with preterm labor with intra-amniotic inflammation. Sci Rep.

[52] Fettweis JM, Serrano MG, Brooks JP, Edwards DJ, Girerd PH, Parikh HI (2019). The vaginal microbiome and preterm birth. Nat Med.

[53] Fawke J, Lum S, Kirkby J, Hennessy E, Marlow N, Rowell V (2010). Lung function and respiratory symptoms at 11 years in children born extremely preterm: the EPICure study. Am J Respir Crit Care Med.

[54] Villamor-Martínez E, Pierro M, Cavallaro G, Mosca F, Kramer B, Villamor E (2017). Probiotic supplementation in preterm infants does not affect the risk of bronchopulmonary dysplasia: a meta-analysis of randomized controlled trials. Nutrients.

[55] Huang J, Zhang L, Tang J, Shi J, Qu Y, Xiong T, Mu D (2019). Human milk as a protective factor for bronchopulmonary dysplasia: a systematic review and meta-analysis. Arch Dis Child Fetal Neonatal Ed.

