# Supplementary material for: Microbial signatures in amniotic fluid at preterm birth and association with bronchopulmonary dysplasia
Source: Respir Res. 2023 Oct 16;24:248. doi: 10.1186/s12931-023-02560-w (PMC10577941; doi:10.1186/s12931-023-02560-w)
Supplement: Supplementary file 1 — Additional file 1: Table S1. List of Amplicon sequence variants (ASV) detected in blanks. Those ASV were considered as potential contaminants and removed from AF dataset before analysis. [file 12931_2023_2560_MOESM1_ESM.docx]

| ASV | phylum | class | order | family | genus |
| --- | --- | --- | --- | --- | --- |
| Asv4388 | *Acidobacteria* | *Acidobacteriia* | *Acidobacteriales* | uncultured | unclassified |
| Asv4988 | *Acidobacteria* | *Subgroup 6* | unclassified | unclassified | unclassified |
| Asv13043 | *Actinobacteria* | *Actinobacteria* | *Corynebacteriales* | *Corynebacteriaceae* | *Corynebacterium 1* |
| Asv13026 | *Actinobacteria* | *Actinobacteria* | *Corynebacteriales* | *Corynebacteriaceae* | *Corynebacterium 1* |
| Asv4356 | *Actinobacteria* | *Actinobacteria* | *Micrococcales* | *Dermabacteraceae* | *Dermabacter* |
| Asv13023 | *Actinobacteria* | *Actinobacteria* | *Micrococcales* | *Dermabacteraceae* | *Dermabacter* |
| Asv81 | *Actinobacteria* | *Actinobacteria* | *Micrococcales* | *Micrococcaceae* | *Paeniglutamicibacter* |
| Asv96 | *Actinobacteria* | *Actinobacteria* | *Micrococcales* | *Micrococcaceae* | *Rothia* |
| Asv3360 | *Actinobacteria* | *Actinobacteria* | *Micrococcales* | *Micrococcaceae* | *Rothia* |
| Asv13018 | *Actinobacteria* | *Actinobacteria* | *Micrococcales* | *Micrococcaceae* | unclassified |
| Asv13027 | *Actinobacteria* | *Actinobacteria* | *Propionibacteriales* | *Nocardioidaceae* | *Nocardioides* |
| Asv36 | *Actinobacteria* | *Actinobacteria* | *Propionibacteriales* | *Propionibacteriaceae* | *Cutibacterium* |
| Asv13028 | *Bacteroidetes* | *Bacteroidia* | *Bacteroidales* | *Muribaculaceae* | unclassified |
| Asv82 | *Bacteroidetes* | *Bacteroidia* | *Flavobacteriales* | *Weeksellaceae* | *Chryseobacterium* |
| Asv614 | *Bacteroidetes* | *Bacteroidia* | *Flavobacteriales* | *Weeksellaceae* | *Cloacibacterium* |
| Asv859 | *Firmicutes* | *Bacilli* | *Bacillales* | *Bacillaceae* | *Bacillus* |
| Asv6 | *Firmicutes* | *Bacilli* | *Bacillales* | *Staphylococcaceae* | *Staphylococcus* |
| Asv150 | *Firmicutes* | *Bacilli* | *Bacillales* | *Staphylococcaceae* | *Staphylococcus* |
| Asv13024 | *Firmicutes* | *Bacilli* | *Bacillales* | *Staphylococcaceae* | *Staphylococcus* |
| Asv9805 | *Firmicutes* | *Bacilli* | *Lactobacillales* | *Aerococcaceae* | *Aerococcus* |
| Asv13025 | *Firmicutes* | *Bacilli* | *Lactobacillales* | *Aerococcaceae* | *Aerococcus* |
| Asv46 | *Firmicutes* | *Bacilli* | *Lactobacillales* | *Lactobacillaceae* | *Lactobacillus* |
| Asv13037 | *Firmicutes* | *Bacilli* | *Lactobacillales* | *Lactobacillaceae* | *Lactobacillus* |
| Asv13041 | *Firmicutes* | *Bacilli* | *Lactobacillales* | *Streptococcaceae* | *Streptococcus* |
| Asv45 | *Firmicutes* | *Bacilli* | *Lactobacillales* | *Streptococcaceae* | *Streptococcus* |
| Asv593 | *Firmicutes* | *Bacilli* | *Lactobacillales* | *Streptococcaceae* | *Streptococcus* |
| Asv550 | *Firmicutes* | *Bacilli* | *Lactobacillales* | *Streptococcaceae* | *Streptococcus* |
| Asv436 | *Fusobacteria* | *Fusobacteriia* | *Fusobacteriales* | *Fusobacteriaceae* | *Fusobacterium* |
| Asv263 | *Proteobacteria* | *Alphaproteobacteria* | *Rhodobacterales* | *Rhodobacteraceae* | *Paracoccus* |
| Asv13022 | *Proteobacteria* | *Deltaproteobacteria* | *Bdellovibrionales* | *Bacteriovoracaceae* | *Peredibacter* |
| Asv13020 | *Proteobacteria* | *Gammaproteobacteria* | *Betaproteobacteriales* | *Burkholderiaceae* | *Janthinobacterium* |
| Asv38 | *Proteobacteria* | *Gammaproteobacteria* | *Enterobacteriales* | *Enterobacteriaceae* | unclassified |
| Asv25 | *Proteobacteria* | *Gammaproteobacteria* | *Pseudomonadales* | *Moraxellaceae* | *Acinetobacter* |
| Asv13021 | *Proteobacteria* | *Gammaproteobacteria* | *Pseudomonadales* | *Moraxellaceae* | *Acinetobacter* |
| Asv21 | *Proteobacteria* | *Gammaproteobacteria* | *Pseudomonadales* | *Pseudomonadaceae* | *Pseudomonas* |
| Table S1  List of Amplicon sequence variants (ASV) detected in blanks. Those ASV were considered as potential contaminants and removed from AF dataset before analysis. | | | | | |
